# Supplementary material for: The genome sequence of the Ribwort slender, Aspilapteryx tringipennella (Zeller, 1839) (Lepidoptera: Gracillariidae)
Source: Wellcome Open Res. 2025 Oct 21;10:588. [Version 1] doi: 10.12688/wellcomeopenres.25049.1 (PMC12848358; doi:10.12688/wellcomeopenres.25049.1)
Supplement: Peer review history

# Reviewer report: Reviewer response for version 1

**DOI:** 10.21956/wellcomeopenres.27615.r144036

**Published:** 2026-01-27

**Authors:** Panagiotis Ioannidis

**Affiliations:** 1 Foundation for Research & Technology - Hellas, Crete, Greece

This paper describes the sequencing and assembly of the genome of the lepidopteran Apilapteryx tringipennella.

 The methodology used for genome sequencing and genome assembly is the one routinely used for all DToL projects and resulted in a very good assembly.

 More specifically, virtually the entire assembly is assigned to one of the 30 chromosome-level scaffolds, thus resulting in an excellent contiguity. However, there are >100 unassigned scaffolds. These scaffolds appear to be of lower-than-expected coverage and of higher-than-expected GC content (Figure 6). It would be nice if the authors could comment on the putative origin of these scaffolds (and, of course, any other features they deem interesting).

 Additionally, all the other assembly evaluation metrics are very good, with k-mer completeness at 99.63%, QV at 59.2. The fraction of complete BUSCOs is at 96.2% and even though it is a good score, it could probably be better. It would also be very nice if the authors could add a brief comment about the BUSCO results.

 Finally, it is good to see that a gene set will be available, even it's not mentioned when exactly. As of today (Jan-26), there's nothing available at the Ensembl website. It is very important to point out that having a gene set is of crucial to most scientists. Genome annotation is a tedious process and not everyone can do it. So, maybe providing a gene set for these DToL assemblies should be almost "mandatory".

Are sufficient details of methods and materials provided to allow replication by others?

Yes

Is the rationale for creating the dataset(s) clearly described?

Yes

Are the datasets clearly presented in a useable and accessible format?

Yes

Are the protocols appropriate and is the work technically sound?

Yes

Reviewer Expertise:

insect genomics, bioinformatics

I confirm that I have read this submission and believe that I have an appropriate level of expertise to confirm that it is of an acceptable scientific standard.

# Reviewer report: Reviewer response for version 1

**DOI:** 10.21956/wellcomeopenres.27615.r138084

**Published:** 2025-11-12

**Authors:** Danilo Trabudo do Amaral

**Affiliations:** 1 Universidade Federal do ABC Centro de Ciencias Naturais e Humanas, Santo André, State of São Paulo, Brazil

*The manuscript is very well prepared, with an excellent assembly and a clear methodological workflow. The text is concise and the results are of high quality. I commend the authors for producing a genome assembly that meets current reference standards.I have only a few recommendations that could further improve clarity and completeness:*

*Please include a short explanation describing the observed asymmetry between the primary and combined k-mer completeness values, in relation to haplotype resolution and the estimated heterozygosity (~2.1%). This will help readers understand why the combined completeness is close to 100% while the primary is around 70%.*

*Since the text already cites synteny with Euspilapteryx, consider adding one line summarizing additional evidence (for example, differential Hi-C coverage, sex-linked read depth, or mapping of male reads) to reinforce the identification of the Z chromosome.*

*Adding a brief paragraph about the repetitive fraction of the genome, optionally including the LTR Assembly Index (LAI), would help contextualize contiguity and N50 results.*

*If applicable, a short mention of whether telomeric or centromeric repeats were detected at chromosome ends would further support the assembly’s chromosomal completeness.*

*Summarize the outcome of the ASCC step in one sentence (e.g., “No scaffolds were flagged as contaminants; X Mb removed prior to the final version”) to improve transparency.*

*Figure 1. The specimen image currently appears of limited morphological quality. Replacing it with a clearer photo or omitting it altogether would strengthen the visual presentation.*

Are sufficient details of methods and materials provided to allow replication by others?

Yes

Is the rationale for creating the dataset(s) clearly described?

Yes

Are the datasets clearly presented in a useable and accessible format?

Yes

Are the protocols appropriate and is the work technically sound?

Yes

Reviewer Expertise:

Bioinformatic

I confirm that I have read this submission and believe that I have an appropriate level of expertise to confirm that it is of an acceptable scientific standard.
